# Posterior Atrophy and Medial Temporal Atrophy Scores Are Associated with Different Symptoms in Patients with Alzheimer’s Disease and Mild Cognitive Impairment

**DOI:** 10.1371/journal.pone.0137121

**Published:** 2015-09-15

**Authors:** Jung-Lung Hsu, Wei-Ju Lee, Yi-Chu Liao, Jiing-Feng Lirng, Shuu-Jiun Wang, Jong-Ling Fuh

**Affiliations:** 1 Graduate Institute of Humanities in Medicine, Taipei Medical University, Taipei, Taiwan; 2 Section of Dementia and Cognitive Impairment, Department of Neurology, Chang Gung Memorial Hospital, Linkou, Taiwan; 3 Brain and Consciousness Research Center, Taipei Medical University, Shuang Ho Hospital, New Taipei City, Taiwan; 4 Neurological Institute, Taichung Veterans General Hospital, Taichung, Taiwan; 5 Faculty of Medicine, National Yang-Ming University Schools of Medicine, Taipei, Taiwan; 6 Institute of Clinical Medicine, National Yang-Ming University Schools of Medicine, Taipei, Taiwan; 7 Institute of Brain Science, National Yang-Ming University Schools of Medicine, Taipei, Taiwan; 8 Brain Research Center, National Yang-Ming University Schools of Medicine, Taipei, Taiwan; 9 Department of Neurology, Neurological Institute, Taipei Veterans General Hospital, Taipei, Taiwan; 10 Department of Radiology, Taipei Veterans General Hospital, Taipei, Taiwan; Johns Hopkins School of Medicine, UNITED STATES

## Abstract

**Background:**

Whether the occurrence of posterior atrophy (PA) and medial temporal lobe atrophy (MTA) was correlated with cognitive and non-cognitive symptoms in Alzheimer’s disease (AD) and mild cognitive impairment (MCI) patients are unclear.

**Methods:**

Patients with probable AD and MCI from a medical center outpatient clinic received attention, memory, language, executive function evaluation and Mini-Mental Status Examination (MMSE). The severity of dementia was rated by the Clinical Dementia Rating (CDR) Sum of Box (CDR-SB). The neuropsychiatric inventory (NPI) subscale of agitation/aggression and mood symptoms was also applied. Magnetic resonance imaging (MRI) was scored visually for the MTA, PA and white matter hyperintensity (WMH) scores.

**Results:**

We recruited 129 AD and 31 MCI (mean age 78.8 years, 48% female) patients. MMSE scores, memory, language and executive function were all significantly decreased in individuals with AD than those with MCI (p < 0.01). MTA and PA scores reflected significant atrophy in AD compared to MCI; however, the WMH scores did not differ. The MTA scores were significantly correlated with the frontal, parieto-occipital and global WMH scores (p < 0.01) while the PA scores showed a correlation with the parieto-occipital and temporal WMH scores (p < 0.01). After adjusting for age, education, APOE4 gene and diagnostic group covariates, the MTA scores showed a significant association with MMSE and CDR-SB, while the right side PA scores were significantly associated with NPI-agitation/aggression subscales (p < 0.01).

**Conclusion:**

Regional atrophy is related to different symptoms in patients with AD or MCI. PA score is useful as a complementary measure for non-cognitive symptom.

## Introduction

Alzheimer’s disease (AD) is the most common form of dementia worldwide. Recently, a nationwide survey in Taiwan showed the age-adjusted prevalence of 8.04% for all type of dementia [1]. In AD, the earliest prominent cognitive symptoms can be characterized as either amnestic or non-amnestic dysfunction [2]. This variable presentation, which reflects the heterogeneity of AD, may be related to the initial dysfunction of the different brain regions as well as additional factors such as genetic or neuropathological findings [3–5]. In addition, 75–90% of AD patients develop “non-cognitive” behavior and psychological symptoms of dementia (BPSD), which are classified into various clusters using the Neuropsychiatric Inventory (NPI) [6–8]. These non-cognitive symptoms have been linked to the dysfunction of different brain structures such as limbic system or cerebral white matter [9–12]. To study the relationships between behavior symptoms and regional brain dysfunction, non-invasive neuroimaging tools, such as brain magnetic resonance imaging (MRI) are extremely helpful.

In structural brain MRI, many automatic computational measurements could be derived, such as regional brain volume, cortical thickness and surface area [13–15]. These measurements allow for an objective evaluation of structural integrity while also permitting the assessment of the entire brain. To gain a precise quantification of brain volume using automatic computational measurements, sophisticated and time-consuming post-imaging analyses are necessary, which may also require specific scanning protocols. In contrast, the visual rating of MR scans can yield sufficient regional anatomical information for both clinical diagnosis and research, with the advantage of speed, flexibility and clinical applicability. Thus, visual rating methods may serve as an appealing alternative to computational methods [16, 17]

Recently-developed visual rating system have been applied to the evaluation of medial temporal lobe atrophy (MTA) and posterior atrophy (PA); these analyses showed that regional atrophy is associated with impairment in specific cognitive domains in AD, providing support for the utility of these method in the diagnostic evaluation of AD [18–21]. These rating scales also had good inter and intra-rater reliability [19, 22]. However, it is unknown whether similar associations may exist between neuropsychiatric, or BPSD symptoms and MTA and PA scores. In the current work, we have endeavored to address this question by examining the relationships between these visual rating scores and clinical symptoms in patients diagnosed with AD as well as those with mild cognitive impairment (MCI).

## Methods

### Participants

Subjects were recruited from the outpatient clinic of Taipei Veterans General Hospital between August 2012 and July 2014. As part of the diagnostic procedure, all patients received a standardized assessment that included dementia history, neuropsychological assessment, laboratory tests and MRI scans. Diagnoses were made during a multidisciplinary consensus meeting. The diagnosis of probable AD followed the clinical criteria for probable AD described by the National Institute on Aging–Alzheimer’s Association [2]. The diagnosis of MCI was made according to the revised 2004 consensus criteria [23]. The cut-off value for a diagnosis of MCI was set to 1.5 SD below the age-adjusted norm for the logical memory test of the Wechsler Memory Scale III (WMS-III)[24]. The logical memory test component of the WMS-III assesses verbal memory by asking the subject to recall two stories immediately following an oral presentation (Part I), and again after a 30-min delay (Part II)[25]. The disease duration was defined as the period between the initial onset of symptom reported by caregiver and the performance of this study. Genomic DNA was isolated from whole blood using the Gentra Puregene kit according to the manufacturer’s protocols (Qiagen, Hilden, Germany). The presence of the ε2, ε3, and ε4 alleles of the APOE gene were determined by assessing the sequences at two SNPs (rs429358 and rs7412) [26, 27]. The APOE4 gene carrier was defined as one of alleles contain ε4 gene.

### Neuropsychological assessment

Cognitive function was assessed using standardized tests that included several domains. The Mini-Mental Status Examination (MMSE) was used to assess global cognition. To evaluate the severity of dementia, the Clinical Dementia Rating (CDR) scores and sum of box (CDR-SB) tests were given with the caregiver input [28–30]. To assess memory, we used the delay recall of 12-items memory test [31, 32]. To examine the language function, we used both a modified Boston Naming Test [33]. A category verbal fluency test was used to evaluate both verbal ability and executive control ability. In the verbal fluency test, the subject named as many fruits as possible within 1 minute [34]. A forward and backward digit span test was used to assess attention and working memory domain [35]. Finally, the Neuropsychiatric Inventory (NPI) questionnaire was administered to assess the frequency and severity of BPSD [36–38]. Two NPI subscale scores were used in this study, namely, NPI-agitation and NPI-mood symptoms subscales [8]. The NPI-agitation/aggression subscale score included agitation/aggression, dis-inhibition, irritability/lability and aberrant motor behavior items from NPI. The NPI-mood subscale score included depression, anxiety and irritability/lability items from NPI. Each subscale score was calculated by the sum of the items’ severity multiplied by its duration.

### MRI and image analysis

**Fig 1:**
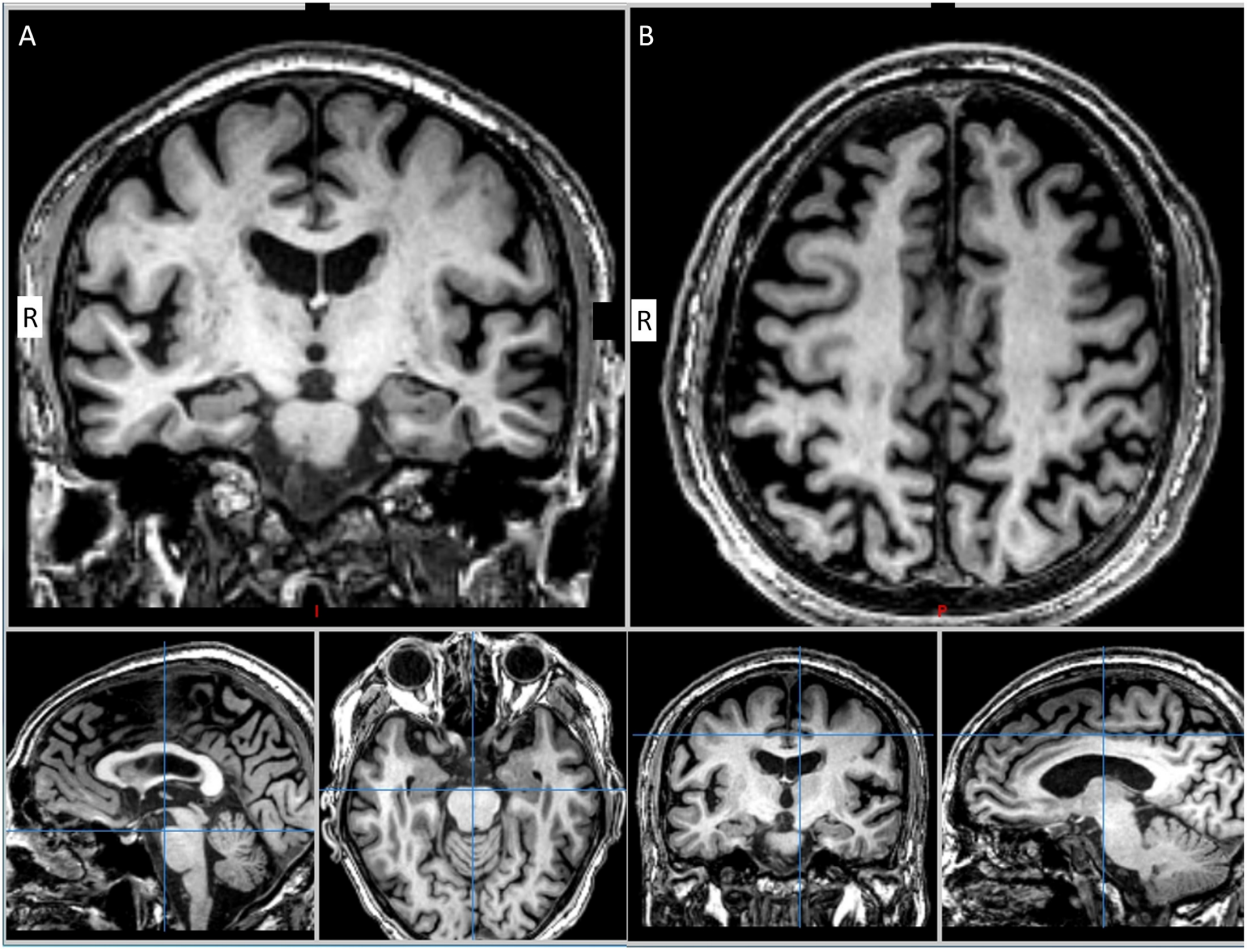
Illustrating the moderate atrophy of MTA and PA scores. (A). the right side MTA score is 2 and the left side MTA score is 1. (B). the both side of PA score is 2. R: right side.

All participants received whole-brain MRI scans (GE, 3T DISCOVERY 750) in the clinical assessment. First, trans-axial T2-weighted scans (TR/TE = 1130/80 ms, NEX = 2, voxel size 0.55 x 0.55 x 10 mm3), 3D fluid-attenuated inversion Recovery (FLAIR) images (TR/TE = 6000/126 ms, inversion time 1861 ms, NEX = 1, voxel size 0.56 x 0.56 x 1 mm3), and high-resolution sagittal T1-weighted images (TR/TE = 9.1/3.7 ms, NEX = 1, voxel size 0.5 x 0.5 x 1.0 mm3) were acquired. The image analysis included a visual rating of MTA and PA on the T1-weighted images. T1-weighted images were viewed in the coronal plane, and MTA scores for the left and right hemispheres were given. MTA was rated on a 5-point scale (0 point, absent; 1 point, minimal; 2 points, mild; 3 points, moderate; and 4 points, severe) based on the height of the hippocampal formation and the width of the choroid fissure and the temporal horn [20]. PA was rated on a 4-point scale (0 point, absent; 1 point, mild sulcal widening and mild atrophy; 2 points, substantial widening and substantial atrophy; and 3 points, end-stage atrophy) based on the posterior cingulate and parieto-occipital sulcus and sulci of the parietal lobes and precuneus [19]. Fig 1 demonstrates the moderate atrophy of MTA and PA scores. The mean scores for MTA and PA from both hemispheres were calculated for statistic analysis. White matter hyperintensity (WMH) was evaluated using a scale of age-related white matter change (ARWMC) scale on both axial T2-weighted image and 3D-FLAIR images [39]. The ARWMC scale rates WMH on a 4-points scale (0 point, no lesions; 1 point, focal lesions; 2 points, beginning confluence of lesions; and 3 points, diffuse involvement of the entire region, with or without involvement of U fibers). A total of five locations (frontal, parieto-occipital, temporal, basal ganglia and infratentorium) were assessed and the sum of both left and right hemisphere ARWMC scores in each location were used for further analysis. All the visual rating scores were evaluated by one of the author (Dr. Hsu). To confirm the consistency of the visual rating scores between investigators, the first 24 AD cases were selected to have both the MTA and PA score evaluated by both Dr. Hsu and Prof. Philip Scheltens [20]. The Ethics Committee and Institutional Review Board of Taipei Veterans General Hospital approved the study. The written informed consent was provided by the participants and their next of kin or legally authorized representatives.

### Statistic analysis

All statistic analyses were performed using SPSS (version 21.0). Independent two sample t-tests and X2 tests were conducted to compare age, gender, neuropsychological tests results, MTA, PA and ARWMC scores between AD and MCI patients. The kappa test was used to assess the inter-rater reliability in visual rating scores. Correlation analysis using Pearson correlation coefficient values was conducted to study the relationship between visual rating scales and age, education, MMSE, CDR-SB and NPI subscales. To assess the relationships between the visual rating scores, cognitive functions and neuropsychiatric symptoms, we used regression analysis with MTA and PA as independent variables, and MMSE, CDR-SB and NPI subscale scores as dependent variables. Age, education, APOE4 gene data and diagnostic group were entered as covariates. Statistic significance was defined as a p-value < 0.01.

## Results

A total 160 patients (probable AD, n = 129; amnestic MCI, n = 31) were recruited during the study period. The average age (mean + standard deviation) was 79.6 + 7.8 years for the AD and 75.4 + 8.6 years for the MCI patients (p = 0.02). Female patients represented 50.4% of the AD and 38.7% of the MCI patients (Table 1). The average disease duration was significant longer in AD group (AD vs. MCI: 41.3 + 48.8 months vs. 26.3 + 21.3 months, p < 0.01). Gender distribution, years of education, and body mass index (BMI) did not differ between the AD and MCI groups. The mean MMSE, delayed recall of 12-item memory test, category verbal fluency and modified Boston naming test were significant lower in the AD than in the MCI groups (p < 0.01). In measurements of non-cognitive symptoms, the AD group displayed significantly higher average subscale scores for NPI-agitation/aggression symptoms and NPI-mood symptoms than did the MCI group. There was also a trend towards a higher proportion of ApoE4 carriers in the AD compared to the MCI group (p < 0.02).

**Table 1:** Demographic and clinical data of AD and MCI patients.

	AD (n = 129)	MCI (n = 31)	*P* value*
Female patients	65 (50.4%)	12 (38.7%)	0.25
Age	79.6 (7.8)	75.4 (8.6)	0.02
Education years	9.9 (4.4)	11.1 (4.7)	0.19
Disease duration (months)	41.3 (48.8)	26.3 (21.3)	**0.01**
BMI	23.7 (3.3)	24.3 (3.9)	0.41
MMSE	18.9 (5.5)	25.5 (3.3)	**< 0.001**
Delayed recall of 12-item Memory test	1.3 (2.0)	4.8 (2.7)	**< 0.001**
Forward digit span	8.7 (2.9)	9.7 (2.4)	0.04
Backward digit span	4.2 (1.9)	5.2 (2.3)	0.03
Category verbal fluency	6.7 (2.9)	9.1 (2.5)	**< 0.001**
Modified Boston naming test	11.5 (2.8)	13.3 (1.9)	**< 0.001**
NPI-agitation/aggression subscale	7.0 (10.9)	1.2 (2.8)	**< 0.001**
NPI-mood subscale	5.4 (8.9)	1.6 (4.3)	**0.001**
CDR			**< 0.001**
0.5	12 (9.3%)	31 (100%)	
1	89 (68.9%)		
2	25 (19.4%)		
3	3 (2.3%)		
ApoE4 carrier	44 (34.1%)	4 (12.9%)	0.02

Values are means with standard deviations unless otherwise indicated. Values in boldface indicate statistically significant differences between groups.

AD, Alzheimer’s disease; MCI, mild cognitive impairment; BMI, body mass index; MMSE, Mini-Mental State Examination; NPI, Neuropsychiatric Inventory; CDR, clinical dementia rating;

* Independent two sample t-tests or Chi-square tests.

### MRI rating

We observed a moderate agreement between the rater and the original author reflected by the inter-rater reliability of the visual rating MTA and PA scores. The Kappa coefficient agreement varies from 0.51 and 0.51 for both hemisphere scores of MTA (p < 0.001 on both side) and 0.57 and 0.43 for both hemisphere scores of PA scores (p < 0.001 on the right side and p = 0.001 on the left side). There was a significantly higher MTA and PA scores for the AD vs. MCI patients. However, for the WMH measurements, the total and the regional ARWMC scores did not differ significantly between the AD and MCI group (Table 2). The MTA and PA scores within each hemisphere did not show a significant correlation (Pearson correlation coefficient: 0.17, p = 0.03 for the right side and 0.13, p = 0.12 for the left side). In terms of anatomical association, we observed significant correlations between the MTA score and the frontal, parieto-occipital and total WMH scores were found in both the AD and the MCI group (Pearson correlation coefficient: 0.36, 0.28 and 0.31 respectively, all p < 0.01). The PA scores also showed a significant association with the parieto-occipital and temporal WMH scores (Pearson correlation coefficient: 0.21 and 0.21, p < 0.01) (Table 3).

**Table 2:** Brain MRI visual rating scores of AD and MCI patients.

	AD (n = 129)	MCI (n = 31)	*P* value*
MTA			
Right	1.9 (0.9)	1.4 (0.9)	**0.01**
Left	2.0 (0.9)	1.5 (0.9)	**0.006**
Mean	1.9 (0.8)	1.4 (0.9)	**0.006**
PA			
Right	1.5 (0.6)	1.1 (0.7)	**0.002**
Left	1.5 (0.7)	0.9 (0.6)	**< 0.001**
Mean	1.5 (0.6)	1.0 (0.6)	**< 0.001**
WMH			
Frontal	2.7 (2.1)	2.5 (2.0)	0.70
Right	1.3 (1.0)	1.3 (1.0)	0.74
Left	1.4 (1.0)	1.3 (1.1)	0.67
Parieto-occipital	2.6 (2.2)	2.4 (2.1)	0.60
Right	1.4 (1.1)	1.2 (1.1)	0.51
Left	1.3 (1.1)	1.2 (1.1)	0.70
Temporal	1.0 (1.7)	0.9 (1.6)	0.65
Right	0.5 (0.9)	0.4 (0.8)	0.46
Left	0.5 (0.9)	0.5 (0.9)	0.87
Basal ganglion	0.1 (0.6)	0.1 (0.4)	0.51
Right	0.1 (0.3)	0.0 (0.2)	0.33
Left	0.1 (0.3)	0.0 (0.2)	0.53
Infratentorium	0.0 (0.2)	0.0 (0.0)	0.18
Right	0.0 (0.2)	0.0 (0.0)	0.08
Left	0.0 (0.1)	0.0 (0.0)	0.32
Total	6.5 (5.5)	5.9 (5.1)	0.53

Values are means with standard deviations. Values in boldface indicate statistically significant differences between groups.

MRI, magnetic resonance imaging; AD, Alzheimer’s disease; MCI, mild cognitive impairment; MTA, medial temporal atrophy; PA, posterior atrophy; WMH, white matter hyperintensity;

* Independent two sample t-test tests.

**Table 3:** Correlation analysis of brain MRI visual rating scores in AD and MCI patients.

	MTA	PA	FWMH	POWMH	TWMH	TOMWH
MTA	-	0.16	0.36*	0.28*	0.18	0.31*
PA		-	0.10	0.21*	0.21*	0.17
FWMH			-	0.78*	0.60*	0.90*
POWMH				-	0.65*	0.92*
TWMH					-	0.82*
TOWMH						-

Values are Pearson correlation coefficients.

MRI, magnetic resonance imaging; AD, Alzheimer’s disease; MCI, mild cognitive impairment; MTA, medial temporal atrophy; PA, posterior atrophy; FWMH, frontal white matter hyperintensity; POWMH, parieto-occipital white matter hyperintensity; TWMH, temporal white matter hyperintensity; TOWMH, total white matter hyperintensity.

*p < 0.01.

We then explored the relationship between the visual rating scores and the clinical parameters that were assessed. MTA scores showed a significant positive association with age and CDR-SB for both the AD and MCI groups. Additionally, MTA scores showed a significant negative correlation with the MMSE scores in both the AD and MCI patients. Only the age and the NPI-agitation/aggression subscale scores were significantly correlated with PA (Table 4).

**Table 4:** Correlation analysis of clinical data and brain MRI visual rating scores in AD and MCI patients.

	Age	Education years	MMSE	CDR-SB	NPI-agitation /aggression	NPI-mood
MTA	0.31*	0.19	-0.30*	0.34*	0.18	0.09
PA	0.40*	0.06	-0.09	0.15	0.26*	0.16
FWMH	0.27*	0.04	-0.14	0.10	0.14	0.13
POWMH	0.32*	0.07	-0.00	0.03	0.17	0.19
TWMH	0.19	0.16	-0.02	0.07	0.22*	0.23
TOWMH	0.28*	0.08	-0.06	0.08	0.20	0.22*

Values are Pearson correlation coefficients.

MRI, magnetic resonance imaging; AD, Alzheimer’s disease; MCI, mild cognitive impairment; MTA, medial temporal atrophy; PA, posterior atrophy; FWMH, frontal white matter hyperintensity; POWMH, parieto-occipital white matter hyperintensity; TWMH, temporal white matter hyperintensity; TOWMH, total white matter hyperintensity; MMSE, Mini-Mental State Examination; NPI, Neuropsychiatric Inventory; CDR-SB, clinical dementia rating sum of boxes.

*p < 0.01.

To further evaluate the relationship between the visual rating scores and the clinical measurements, we performed a multivariate regression analysis. Following adjustments for age, education, APOE4 gene and diagnostic group, the MMSE was still significantly correlated with MTA scores. We also observed a significant association of the CDR-SB score with MTA. PA scores, in contrast, did not exhibit any significant correlation with MMSE and CDR-SB scores following adjustments for age and education covariates (p = 0.56 and P = 0.28). The PA, but not the MTA, score show a trend of association with the NPI-agitation/aggression subscale values following adjustment for age (p = 0.03 for PA vs. P = 0.18 for MTA). We further separated the right and left hemisphere PA scores to perform the regression study. In this regression analysis, the right hemisphere PA scores showed a higher R-square value than did the left hemisphere scores (R-square = 0.36, p = 0.003 (right) vs. R-square = 0.29, p = 0.19 (left); Table 5).

**Table 5:** Summary of regression analyses for variables predicting the MMSE, CDR-SB scores and subscale of NPI-agitation scores in AD and MCI patients.

Variables entered	R	R^2^	R^2^ Change	F Change	df
MMSE as dependent variable					
Block 1. Age, education years, APOE4 carrier and diagnostic group	0.49	0.24	0.24	11.97*	155
Block 2. MTA*	0.55	0.30	0.07	14.93*	154
CDR-SB as dependent variable					
Block 1. Age, education years, APOE4 carrier and diagnostic group	0.52	0.27	0.27	14.01*	155
Block 2. MTA*	0.57	0.32	0.05	12.27*	154
NPI-agitation/aggression as dependent variable					
Block 1. Age, education years, APOE4 carrier and diagnostic group	0.28	0.08	0.08	3.25	155
Block 2. PA (right side)*	0.36	0.13	0.05	8.79*	154

MMSE, Mini-Mental State Examination; NPI, Neuropsychiatric Inventory; CDR-SB, clinical dementia rating sum of boxes; AD, Alzheimer’s dementia; MCI, mild cognitive impairment; MTA, medial temporal atrophy visual rating score.

*p < 0.01

## Discussion

In this study, we used a method of simple visual rating to evaluate the association between morphological brain features and clinical symptoms. We show that MTA scores are correlated with performance on the MMSE and CDR-SB scores, measures of cognition, in AD and MCI patients, whereas PA scores were associated with scores on the NPI-agitation/aggression subscale. These findings provide support for the idea that regional atrophy in different brain structure may be intimately related to cognitive and non-cognitive symptoms in AD and MCI. Moreover, we provide the first evidence that the degree of PA score correlates with non-cognitive symptoms, as measured by the NPI subscales. Our findings further show that the application of visual rating scores (both MTA and PA) is useful for the evaluation of AD and MCI patients in clinical studies.

The MTA score had been in use since 1992, and it is one of the widely-used visual rating systems used in clinical and research field [20, 40]. Evidence for MTA has previously been used to support the diagnosis of AD [20, 41]. In studies of cognition, the MTA score has been shown to correlate with both memory and executive function [42–44]. In previous studies, the degree of MTA has also been applied to predict the conversion from MCI to AD [45, 46]. The MTA score may be influenced by the age and WMH but it appears to be independent of the PA score, at least in AD patients [19, 47, 48]. Consistent with previous findings, our current study found that MTA scores were correlated with age (Pearson correlation coefficient = 0.31, p < 0.01) but not with PA scores (Pearson correlation coefficient = 0.16, p = 0.04). In addition, MTA scores showed a significant correlation with both global and regional WMH scores (both frontal and parieto-occipital), in line with previous literatures [47–49].

The PA score is a recently developed visual rating scale that has been shown to be able to discriminated AD from dementia with Lewy bodies and frontotemporal lobar degeneration [19]. Furthermore, PA ratings can distinguish between younger control subjects and early-onset AD, but not between older control subjects and late-onset AD [21]. AD patients with higher PA scores have been shown to have worse performance on tasks of visuospatial and executive function [18]. In the current work, we observed that PA scores showed a significant correlation with posterior regional WMH (parieto-occipital and temporal regions) in AD and MCI patients. This anatomical association may reflect concomitant regional gray matter and white matter changes in the brain. We did not find any significant association between PA scores and digit backward and category verbal fluency test in both AD and MCI patients (p = 0.18 and p = 0.33). In addition, only a trend was observed for a correlation between the PA scores and the digit backward in AD group (p = 0.02).

In tests of non-cognitive symptoms, we found that the mean PA scores had a trend of association with scores on the NPI-agitation/aggression subscale (p = 0.03). More specifically, we found that PA scores in the right hemisphere, but not the left, were significantly associated with the NPI-agitation/aggression subscale. In addition to the PA, we observed that the temporal WMH scores is significant correlated with scores of the NPI-agitation/aggression subscale. Symptoms of agitation/aggression have previously been shown to be associated with brain structure such as lateral frontal, parietal, temporal and anterior cingulate gyrus in AD or MCI patients [12, 50–53]. Another study showed that right fronto-temporal dysfunction may be linked to aggression behavior in dementia patients [54]. In a functional MRI study, Kumari et al found that bilateral frontal and right inferior parietal lobe activity was associated with aggression behavior in schizophrenia patients [55, 56]. From previous studies, the right hemisphere rather than left hemisphere may have stronger association with aggression behavior [54, 56]. Our results demonstrate that both right PA and temporal WMH scores have a significant association with ratings on the NPI-agitation/aggression subscale in both AD and MCI patients, which has not been reported before.

### Limitation

There had some limitations to the current work. First, we have no postmortem data from the individuals that comprise our dataset. Therefore, the possibility of misdiagnosis cannot be ruled out. Nevertheless, we used the standardized workup procedure and all patients fulfilled the criteria of probable AD and MCI. These standardized measures may help to reduce the potential likelihood of the misclassification of patients. Secondary, only two atrophy measures were used in the current study and the PA score was measured from a large aggregate of regions, therefore the finer localization was not possible. Both MTA and PA are the areas that are more commonly affected in AD than MCI. The kappa values of interrater reliability of MTA and PA in the current study are around 0.5, which is in moderate agreement. It might influence the study findings. We included the APOE4 gene data and diagnostic group as covariates in multivariate regression analysis to decrease the confounding effect. Third, the irritability/lability from NPI items were included in both NPI-agitation and NPI-mood symptoms subscales according our prior study. The multivariate regression analysis between irritability scores and PA scores is non-significant. It needs further study to confirm this finding. Additionally, we did not perform an extensive cognitive examination that included assessments of executive, visuospatial and praxis function. The category verbal fluency is more related to measure the semantic memory function then executive function [57]. The lack of such data may restrict the interpretation of our study as to the relationship between the PA score and more subtle aspects of cognition function. A great strength of the current study, we believe, is the inclusion of the NPI assessment to explore non-cognitive symptoms in MCI and AD patients, and how these may relate to aspects of brain structure.

## Conclusion

Our results showed that, in MCI and AD patients, data gained by the visual rating of MTA and PA scores are not a reduplication data; rather they provide complementary information relevant to both cognitive and non-cognitive symptoms. In addition, we find that the visual rating of PA scores provides additional relevance to symptoms of agitation. Further study, with a large patient sample, combined with a more detailed battery of neuropsychological tests, will be necessary to confirm these findings.
